# Sequence variants in *HTRA1* and *LOC387715/ARMS2* and phenotype and response to photodynamic therapy in neovascular age-related macular degeneration in populations from Israel

**Published:** 2008-12-08

**Authors:** Itay Chowers, Tal Meir, Michal Lederman, Nitza Goldenberg-Cohen, Yoram Cohen, Eyal Banin, Edward Averbukh, Itzhak Hemo, Ayala Pollack, Ruth Axer-Siegel, Orly Weinstein, Josephine Hoh, Donald J. Zack, Tural Galbinur

**Affiliations:** 1 Department of Ophthalmology, Hadassah-Hebrew University Medical Center, Jerusalem, Israel; 2 Department of Ophthalmology, Rabin Medical Center, Petah Tiqva, Israel; 3 Cancer Research Center, Sheba Medical Centre, Tel Aviv University, Tel Aviv, Israel; 4 Department of Ophthalmology, Kaplan Medical Center, Rehovot, Israel; 5 Department of Ophthalmology, Soroka University Medical Center, Beer Sheva, Israel; 6 Department of Epidemiology and Public Health, Yale University, New Haven, CT; 7 Wilmer Eye Institute, Johns Hopkins University School of Medicine, Baltimore, MD

## Abstract

**Purpose:**

Single nucleotide polymorphisms (SNPs) in the tightly linked *LOC387715/ARMS2* and *HTRA1* genes have been associated with age-related macular degeneration (AMD). We tested whether these SNPs are associated with AMD in Israeli populations, if they underlie variable phenotype and response to therapy in neovascular AMD (NVAMD), and if *HTRA1* expression in vivo is associated with its promoter variant.

**Methods:**

Genotyping for the rs10490924 SNP in *LOC387715/ARMS2* and the rs11200638 SNP in *HTRA1* was performed on 255 NVAMD patients and 119 unaffected controls from Ashkenazi and Sephardic Jewish, and from Arab origins which are the main ethnic groups composing the Israeli population. Genotyping was correlated with phenotype and response to therapy among 143 patients who underwent photodynamic therapy (PDT). *HTRA1* mRNA levels in white blood cells (WBCs), measured by quantitative PCR, were correlated with genotype in 27 participants.

**Results:**

Both SNPs were in almost complete linkage disequilibrium (D'=0.96–1). Homozygotes for the T allele of rs10490924 had an odds ratio (OR) of 8.6, with a 95% confidence interval (CI) of 3.5–20.8, and homozygotes for the A allele of rs11200638 had an OR of 10.7, with a 95% CI of 3.2–35.7, for having AMD (p<0.00001). There was no association among these SNPs and phenotype or response to PDT. *HTRA1* mRNA levels in WBCs were not associated with rs11200638 genotypes.

**Conclusions:**

The rs10490924 SNP in *LOC387715/ARMS2* and the rs11200638 SNP in *HTRA1* are strongly associated with NVAMD in this Israeli population. These variants do not have a major contribution to the variable phenotype and response to PDT which characterize NVAMD.

## Introduction

Single nucleotide polymorphisms (SNPs) in chromosome 10q26 are associated with the risk for having age-related macular degeneration (AMD) in several populations [1-18]. Since several 10q26 SNPs are associated with disease risk, it has been difficult to determine if the T allele of rs10490924 located in the coding region of the *LOC387715/ARMS2* (age-related maculopathy susceptibility 2) gene, or the A allele of rs11200638 located in the promoter region of high-temperature requirement factor A1 (*HTRA1*) have a role in the pathogenesis of the disease [6-9,13,19].

The *HTRA1* gene encodes a heat shock serine protease which is expressed in the retina and can regulate transforming growth factor-β (TGF-β) signaling [7,20]. Evidence has conflicted with respect to the correlation between rs11200638 genotypes and *HTRA1* expression levels [6-8].

The *LOC387715/ARMS2* gene encodes a putative 12 kDa protein. Although it has been suggested that *LOC387715/ARMS2* may not actually encode a protein, recent data has shown that it encodes a mitochondrial outer membrane protein that is also expressed in the retina [6]. The T allele of rs10490924 changes amino acid 69 from alanine to serine (A69S variant) in the putative *LOC387715/ARMS2* protein [6,13]. Fritsche [21] and colleagues recently described a related variant in this gene that is associated with AMD and results in rapid mRNA turnover and undetectable expression levels in homozygous carriers.

Neovascular AMD (NVAMD) shows variable phenotype in terms of several parameters, including age of onset, neovascular lesion type and size, and response to treatment. Efforts are underway to identify genetic and other biomarkers that correlate with these various disease parameters. Of particular interest would be the identification of pharmacogenetic markers that might be able to predict response to therapy, since such information could potentially be used to guide choice, and perhaps frequency, of treatment for the individual patient. Recently, a genetic variant in the complement factor H (*CFH*) gene, which is strongly associated with AMD, has been reported to be associated with neovascular lesion type, size, and response to photodynamic therapy (PDT) and injections of the antivascular endothelial growth factor compound bevacizumab [22-26]. The *HTRA1* polymorphism was associated with classic lesion type [26]. However, similar associations have not been consistently found in other populations [10,27,28].

To test the association of the 10q26 SNPs with NVAMD in Israeli populations and to expand our knowledge about phenotype-genotype correlation, we first evaluated *HTRA1* and *LOC387715/ARMS2* polymorphisms and NVAMD risk in Israel. The study included Arabs, Sephardic Jews, and Ashkenazi Jews which are the main ethnic groups composing the Israeli population. After finding a significant association, similar in magnitude to that reported in other populations, we assessed if these SNPs could account for some of the observed variation in clinical characteristics and response to PDT among the NVAMD patients. Finally, as another approach to explore whether the SNP in the 5′-upsteam region of the *HTRA1* promoter influences promoter activity, we tested to see if there was a correlation between genotype and *HTRA1* mRNA levels in vivo as measured in the white blood cells (WBCs) of patients and controls.

## Methods

### Patients and genotyping

The study included 255 NVAMD patients recruited from four retina clinics in Israel and 119 unaffected controls (from the department of Ophthalmology) who were evaluated for routine eye examination or for pathologies other than AMD, in the Department of Ophthalmology of the Hadassah – Hebrew University Medical Center in Jerusalem, Israel. Institutional Ethics Committee approval was obtained for the study, and each patient signed an informed consent form. AMD was diagnosed and graded according to the age related eye disease study (AREDS) trial classification [29]. Inclusion criteria for the control group included age over 60 years, clear cornea, lens, and vitreous which enabled ophthalmoscopy, and absence of intermediate size drusen, multiple small drusen, or retinal pigment epithelial abnormalities characteristics of AMD (AREDS category I). The data set was described in a manuscript evaluating *CFH* variants in the Israeli population [30]. Briefly, female:male ratio was balanced between AMD patients and controls. Mean age in the controls (70.8±8.2) was lower than that of AMD patients (78.1±7.6; p<0.05, unpaired, two-sided, *t*-test). Median follow-up of NVAMD patients having PDT was 16 months (range 1–156 months).

WBCs were separated from blood samples from 27 individuals included in the genotyping study (22 NVAMD patients and 5 unaffected controls). These samples were used for RNA extraction. For WBC separation, 4 CC of whole blood were drawn and shipped to the laboratory on wet ice. RNA was then extracted as described below.

The Israeli population is composed of several ethnic groups, which include Ashkenazi Jews, Sephardic Jews, and Arabs. Studies showed that these subpopulations may be genetically defined, for example, by polymorphisms and microsatellite loci on the Y chromosome [31]. Intermarriage between the groups was uncommon among parents of the elderly individuals included in the study. The control group included 10 Arabs, 40 Sephardic Jews, and 68 Ashkenazi Jews, while the study group included 11 Arabs, 75 Sephardic Jews, and 163 Ashkenazi Jews (p=0.13, χ^2^ test). The ethnicity of one individual from the control group and 6 NVAMD patients was unknown.

Genotyping for the rs11200638 SNP in the putative promoter region of *HTRA1* and for the rs10490924 SNP in the coding region of the *LOC387715/ARMS2* gene was performed. This was done by sequencing PCR products containing these SNPs (Table 1).

**Table 1:** Primers used for genotyping and QPCR.

**Primer**	**Sequence**
Primers for genotyping	
*LOC387715/ARMS2 *forward	TACCCAGGACCGATGGTAAC
*LOC387715/ARMS2 *reverse	GAGGAAGGCTGAATTGCCTA
*HTRA1* forward	CGGATGCACCAAAGATTCTCC
*HTRA1* reverse	TTCGCGTCCTTCAAACTAATGG
Primers for QPCR	
*HTRA1* forward	AGCTGGGACTTCGGAACTCC
*HTRA1* reverse	TCCGGAATTTCCATAATTGATGA
GAPDH forward	GGGGGAGCCAAAAGG GTCAT
GAPDH reverse	GCCCCAGCGTCAAAGGTGGA

Sequence of primers which were used for genotyping of *LOC387715/ARMS2* (rs10490924) and HTRA1 (rs11200638) SNPs and for QPCR for assessment of HTRA1 mRNA levels.

Detailed retrospective clinical information was available on a subgroup of 143 sequential NVAMD patients (of the 255 patients enrolled in the study) who were treated with PDT at the Hadassah–Hebrew University Medical Center. These patients were included in the phenotype-genotype analysis and for the purpose of analysis eyes of these patients which underwent PDT were considered as the study eye. Fluorescein angiograms were reviewed by retina specialists (I.C., E.B., I.H., and E.A.) who were masked with respect to genotype results; choroidal neovascularization was classified as classic, predominantly classic, minimally classic, or occult following the guidelines of the Macular Photocoagulation Study Group [32]. Retinal angiomatous proliferation (RAP) was classified as occult lesions. Review of the entire group of patients was also performed in a masked fashion (with respect to previous lesion type classifications and to genotypes) by one of the investigators (I.C.). There was agreement in 83.4% of cases between this investigator and the classification by the treating retina specialist (kappa measurement of agreement=0.68, p<0.0001). Classification of lesion type according to the observer who reviewed the entire group of patients was used for statistical analysis. The standard PDT protocol for NVAMD was applied [33]. Briefly, patients received 6 mg/m^2^ body surface area of verteporfin (Visudyne; Novartis Ophthalmics, Hettlingen, Switzerland) intravenously over 10 min. Fifteen min after commencement of the infusion exposure of 50 J/cm^2^ was applied at the radiant level of 600 mW/cm^2^ over 83 s using a diode laser (Visulas s 690, Zeiss, Switzerland), and the PDT laser lens (Volk, Mentor, OH).

The association between smoking and the risk for having NVAMD, and its interaction with the 10q26 SNPs was analyzed. Individuals were considered as smokers if they were smoking at the time of the study or if they smoked in the past. Individuals who never smoked were considered as non-smokers.

### Quantitative real-time RT–PCR

*HTRA1* mRNA levels were measured in WBCs from 27 participants (22 NVAMD patients and 5 unaffected controls older than 60 years of age). WBCs were separated from whole blood using hypotonic lysis buffer containing 155 mM NH_4_Cl (Gadot, Or Akiva, Israel), 10 mM CH_2_O_3_·NH_3_ (Sigma-Aldrich, St. Louis, MO), and 0.1 mM EDTA (pH 7.4; J.T. Baker, Philipsberg, NJ). Next, 8 ml of buffer was added to 4 ml of blood, which was then stored on ice for 10 min and centrifuged at 2,000x g at 4 °C for 10 min. Supernatant was discarded and the sequence was repeated an additional time. The WBC pellet was resuspended in 1 ml of TRI Reagent (Sigma). RNA was then extracted followed by treatment with DNase (DNAfree; Ambion, Austin, TX), and cDNA was synthesized from 1 ug of total RNA using the Reverse iT 1st Strand Synthesis Kit (ABgene, Epsom, UK) and oligo dT primers. Quantitative real-time RT–PCR (QPCR) was performed with *HTRA1* specific primers (Table 1) using the ABI Prism 7000 SDS instrument (Applied Biosystems, Foster City, CA). Measurements of *GAPDH* were used for normalization of expression levels across samples.

### Statistical analysis

Statistical analysis and power calculations were performed using SPSS (SPSS, Chicago, IL) and Instat software (GraphPad, San Diego, CA) as we have previously described [30]. Briefly, logistic regression and χ^2^ tests were applied to assess odds ratios, confidence intervals, and significance. Linkage disequilibrium was assessed by D’ calculation. Based on the number of individuals included in the analysis this study had 85% power for identification of an association between genotypes and lesion type in the magnitude described by Brantley and colleagues [22]. The study also had 94% power for identification of an association between genotypes and visual acuity following PDT in the magnitude, which was described by the same group [25].

## Results

### Association of 10q26 SNPs with NVAMD

Both the *HTRA1* rs11200638 SNP and the *LOC387715/ARMS2* rs10490924 SNP complied with Hardy–Weinberg equilibrium. Genotypes for both SNPs were in almost complete linkage disequilibrium, with D' values of 1.00 for NVAMD patients and 0.96 for controls. Distribution of the genotypes for both SNPs was significantly different between NVAMD patients and controls (p<0.0001; Table 2 and Table 3).

**Table 2:** Frequency of *LOC387715/ARMS2* (rs10490924) alleles and genotypes in NVAMD patients and unaffected controls.

***LOC387715/ARMS2* (rs10490924)**	**AMD**	**Unaffected**	**p**	**OR** **(95% CI)**
By allele				
Entire population (G/T; %)*	279/219 (56/44)	190/48 (80/20)	<0.0001	3.1 (2.2–4.5)
Ashkenazi Jews	189/129 (59.4/40.6)	110/26 (80.8/19.2)	<0.0001	2.9 (1.8–4.7)
Sephardic Jews	76/72 (51.4/48.6)	63/17 (78.8/21.2)	<0.0001	3.5 (1.9–6.6)
Arabs	8/12 (40/60)	16/4 (80/20)	0.022	6 (1.5–24.7)
By genotype				
Entire Population			<0.0001	
GG (%)	91 (36.5)	77 (64.2)		
TG (%)	97 (39)	36 (30.3)	0.0012	2.3 (1.4–3.7)
TT (%)	61 (24)	6 (5)	<0.0001	8.6 (3.5–20.8)
Ashkenazi Jews			<0.0001	
GG (%)	67 (42.1)	45 (66.2)		
TG (%)	55 (34.6)	20 (29.4)	0.17	1.6 (3.1–0.82)
TT (%)	37 (23.3)	3 (4.4)	0.003	9.7 (41.6–2.2)
Sephardic Jews			0.0003	
GG (%)	20 (27)	26 (65)		
TG (%)	36 (48.6)	11 (27.5)	0.003	4.3 (10.4–1.7)
TT (%)	18 (24.3)	3 (7.5)	0.001	7.8 (30.3–2)
Arabs#			0.036	
GG (%)	3 (30)	6 (60)		
TG (%)	2 (20)	4 (40)		
TT (%)	5 (50)	0 (0)		

Comparison of the frequency (%) of the *LOC387715/ARMS2* (rs10490924) variant between NVAMD patients and controls in the entire Israeli population and in the Ashkenazi, Sephardic, and Arab subpopulations. Increased prevalence of the T variant was associated with the disease in the entire population as well as in Ashkenazi, Sephardic, and Arab subpopulation. CI- confidence interval, OR- odds ratio. The asterisk indicates reliable genotyping for rs10490924 was obtained from 249 of the 255 patients and for each of the 119 controls. The hash mark represents there were too few Arabs to obtain reliable ORs and CIs for analysis of genotypes in this sub population.

**Table 3:** Frequency of *HTRA1* (rs11200638) alleles and genotypes in NVAMD patients and unaffected controls

***HTRA1*** **(rs1200638)**	**AMD**	**Unaffected**	**p**	**OR (95% CI)**
By allele				
Entire population (G/A; %)	325/185 (63.7/36.3)	197/41 (82.8/17.2)	<0.0001	2.7 (1.9–4)
Ashkenazi Jews	212/114 (65/35)	114/22 (83.8/16.2)	<0.0001	2.8 (1.7–4.6)
Sephardic Jews	95/55 (63/37)	65/15 (81.3/18.7)	0.0065	2.5 (1.3–4.8)
Arabs	10/12 (45.4/54.6)	17/3 (0.85/0.15)	0.01	6.8 (1.5–30.1)
By genotype				
Entire population			<0.0001	
GG (%)	116 (45.5)	81 (68.1)		
GA (%)	93 (36.5)	35 (29.4)	0.013	1.8 (1.1–3.0)
AA (%)	46 (18.0)	3 (2.5)	<0.0001	10.7 (3.2–35.7)
Ashkenazi Jews			0.011	
GG (%)	80 (49.1)	48 (70.6)		
GA (%)	52 (31.9)	18 (26.5)	0.24	1.5 (3–0.76)
AA (%)	31 (19)	2 (2.9)	0.009	7.2 (31.2–1.6)
Sephardic Jews			0.02	
GG (%)	30 (40)	26 (65)		
GA (%)	35 (46.7)	13 (32.5)	0.044	2.3 (5.3–1.02)
AA (%)	10 (13.3)	1 (2.5)	0.046	8.7 (71.5–1.04)
Arabs#			0.05	
GG (%)	4 (36.4)	7 (70)		
GA (%)	2 (18.1)	3 (30)		
AA (%)	5 (45.5)	0 (0)		

Comparison of the frequency (%) of the HTRA1 (rs11200638) variant between NVAMD patients and controls in the entire Israeli population and in the Ashkenazi, Sephardic, and Arab subpopulations. Increased prevalence of the A variant was associated with the disease in the entire population as well as in Ashkenazi, Sephardic, and Arab subpopulation. CI- confidence interval, OR- odds ratio. The hash mark represents there were too few Arabs to obtain reliable ORs and CIs for analysis of genotypes in this sub population.

Homozygotes for the T allele of rs10490924 (*LOC387715/ARMS2*) had an odds ratio (OR) of 8.6 with a 95% confidence interval (CI) of 3.5–20.8, while heterozygotes had an OR of 2.3 (95% CI of 1.4–3.7) for having NVAMD compared with homozygotes for the wild-type allele (Table 2). Combined, individuals either homozygous or heterozygous for the T allele had an OR of 3.2 (95% CI of 2–5; p<0.0001) compared with individuals homozygous for the G allele for having AMD. Analysis according to allele distribution showed similar findings to analysis according to genotypes for each of the SNPs (Table 2).

Homozygotes for the A allele of rs11200638 had an OR of 10.7 (95% CI of 3.2–35.7), while heterozygotes had an OR of 1.8 (95% CI of 1.1–3) for having NVAMD compared with homozygotes for the wild-type allele (Table 3). Combined, individuals either homozygous or heterozygous for the A allele had an OR of 2.5 (95% CI of 1.6–4; p<0.0001) compared with individuals homozygous for the G allele for having AMD.

Subgroup analysis was performed to evaluate for association of both SNPs with NVAMD among Ashkenazi Jews, Sephardic Jews, and Arabs. The rs10490924 (*LOC387715/ARMS2*) SNP was associated with NVAMD among Ashkenazi Jews (p<0.0001, χ^2^ test), Sephardic Jews (p=0.0003, chi-square test), and Arabs (p=0.036, χ^2^ test). Analysis according to allele distribution showed similar findings (Table 2). The rs11200638 (*HTRA1*) SNP was also associated with NVAMD among Ashkenazi Jews (p=0.011, χ^2^ test), Sephardic Jews (p=0.044, χ^2^ test), and Arabs (p=0.05, χ^2^ test). Analysis according to allele distribution showed similar findings (Table 3).

Smoking is an established risk factor for AMD. Since this risk factor was also associated with chromosome 10q26 SNPs in other populations [11], we evaluated its effect in our population. Smoking was associated with the risk for having AMD (OR of 3; 95% CI of 1.7–5.3; p<0.001) in the entire population; however, logistic regression found no interactions between smoking and either homozygosity (p=0.359) or heterozygosity for the risk allele of rs10490924. There were also no interactions between smoking and either homozygosity (p=0.99) or heterozygosity (p=0.29) for the risk allele of rs11200638.

### Association of 10q26 SNPs with phenotype of NVAMD

Following the establishment of an association among the *HTRA1* rs11200638 SNP, the *LOC387715/ARMS2* rs10490924 SNP, and NVAMD in the Israeli population, we explored possible correlations between the genotypes and clinical characteristics of NVAMD and response to PDT. This analysis included 143 sequential NVAMD patients who were treated in the Department of Ophthalmology of the Hadassah Medical Center and who were characterized in terms of phenotype and response to PDT. Reliable genotyping for rs11200638 was obtained from all of these participants and for rs10490924 from 139 of these patients.

There was no significant association between these SNPs and gender, history of smoking, lesion type, initial and final visual acuity, and number of PDT sessions required. The T allele of rs10490924 was associated with a positive family history for AMD while the A allele of rs11200638 showed a trend toward such an association (Table 4 and Table 5). While 23.7% of individuals carrying at least one risk allele (A) of rs11200638 had a positive family history for AMD, only 8.6% of the individuals homozygous to the wild-type allele had a positive family history (p=0.043, χ^2^ test). However, this p value would lose significance if any form of multiple hypothesis testing correction was applied. Similarly, while 22.8% of individuals carrying at least one risk allele (T) of rs10490924 had positive family history for AMD, only 4.4% of the individuals homozygous for the wild-type allele had a positive family history (p=0.008, chi square test). The risk alleles of both SNPs showed a trend toward an association with younger age of onset of NVAMD and with larger lesion size (Table 4  and 5).

**Table 4:** Correlation among demographic and phenotypic characteristics of NVAMD and genotyping for *LOC387715/ARMS2* (rs10490924).

	***LOC387715/ARMS2* (rs10490924) genotype**			
	**TT**	**TG**	**GG**	**p**
Gender (female/male)	21/14	24/27	20/33	0.12
Lesion type* (classic/occult)	11/24	23/28	19/34	0.4
Family History of AMD (yes/no) ^#^	9/19	7/35	2/43	0.006
Age (mean±SD, in years)	76.4±8	78.9±8.5	80.1±7.2	0.1
Smoking (yes/no) ^#^	12/20	25/25	27/25	0.42
Initial VA (mean±SD, logMAR)	1.06±0.83	0.94±0.68	1.14±0.79	0.44
Lesion size (mean±SD, in µm)	4081±1347	3669±1265	3623±1288	0.34
Number of PDT sessions	2.2±2.2	2.6±1.9	2±1.6	0.22
Final VA (mean±SD, logMAR)	1.5±0.9	1.51±0.83	1.37±0.93	0.67

The asterisk indicates that classic lesions included predominantly classic and pure classic lesions. Occult lesions included minimally classic and occult lesions. Analysis according to four lesion types showed similar results. # Reliable information on family history was obtained from 117 out of the 143 participants included in this analysis, and smoking history was obtained from 138 of the 143 study participants.

**Table 5:** Correlation among demographic and phenotypic characteristics of NVAMD and genotyping for *HTRA1* (rs11200638)

	***HTRA1* (rs11200638) genotype**			
	**AA**	**GA**	**GG**	**p**
Gender (female/male)	14/10	25/25	29/40	0.35
Lesion type* (classic/occult)	7/17	22/28	26/43	0.46
Family History of AMD (yes/no) ^#^	5/14	9/31	5/53	0.08
Age (mean ± SD, in years)	75.6±7	79.2±8.5	79.5±7.8	0.1
Smoking (yes/no) ^#^	11/12	22/26	33/34	0.93
Initial VA (mean ± SD, logMAR)	1.1±0.8	0.928±0.7	1.1±0.8	0.38
Lesion size (mean ± SD, in µm)	4250±1261	3696±1290	3603±1261	0.17
Number of PDT sessions	2.5±1.9	2.4±1.8	2.2±1.9	0.78
Final VA (mean ± SD, logMAR)	1.6±0.84	1.5±0.84	1.36±0.94	0.45

The asterisk indicates classic lesions included predominantly classic and pure classic lesions. Occult lesions included minimally classic and occult lesions. Analysis according to four lesion types showed similar results. # Reliable information on family history was obtained from 117 out of the 143 participants included in this analysis, and smoking history was obtained from 138 of the 143 study participants.

### mRNA levels of *HTRA1* in WBCs from NVAMD patients

**Figure 1:**
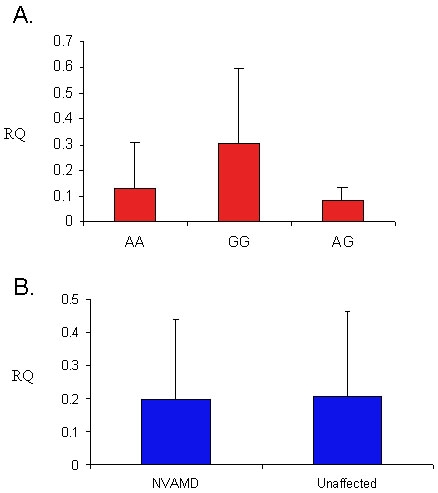
Relative expression levels of *HTRA1* in NVAMD patients and unaffected controls. **A:** Shown are expression levels in white blood cells according to rs11200638 genotypes in individuals homozygous for the wild-type allele (GG, n=13), participants homozygous for the risk allele (AA, n=6), and in heterozygous participants (AG, n=8). Differences between the three groups were not significant (p>0.1 for each comparison). **B:** Shown are mRNA levels of *HTRA1* in 22 NVAMD patients and 5 unaffected individuals (p=0.9). In both **A** and **B**, RQ represents relative HTRA1 mRNA levels. Error bars represent standard deviation (SD).

mRNA levels of *HTRA1* were measured using QPCR in WBCs from 22 NVAMD patients and 5 unaffected controls to assess the effect of the *HTRA1* promoter variant on *HTRA1* expression. ANOVA showed no significant differences in *HTRA1* mRNA levels among individuals with different rs11200638 genotypes (Figure 1A), and between patients and controls (Figure 1B). HTRA1 expression levels were similar in homozygote of the A allele and homozygote of the G allele (p=0.2).

## Discussion

This study established an association between the A allele of rs11200638, located in the putative promoter region of *HTRA1*, and the T allele of rs10490924 (A69S variant), located in the coding region of *LOC387715/ARMS2*, and NVAMD in the Israeli population. Both SNPs are strongly associated with NVAMD among Ashkenazi and Sephardic Jews. While NVAMD is relatively uncommon among Arabs in Israel [34], our data suggest that 10q26 SNPs are also associated with NVAMD in this ethnic group. Similar to findings from fair-skinned populations and Japanese populations, these SNPs are in almost complete linkage disequilibrium in the Israeli populations [1,7,8].

Increased prevalence of the same *HTRA1* and *LOC387715/ARMS2* variants among AMD patients was described in several fair-skinned populations as well as in cohorts from India, Japan, and China [1-8,21,35-39]. The magnitude of the association between these SNPs and NVAMD in Israel is similar to the one which was reported in other populations. For example, Rivera and colleagues [13] found an OR of 2.7 for AMD patients who were heterozygous for the *LOC387715/ARMS2* A69S variant and an OR of 8.2 for patients who were homozygous for it . In the Israeli population participants who were heterozygous for this variant had an OR of 2.3 while those homozygous for it had an OR of 8.6 for having AMD. A stronger linkage of these SNPs with neovascular AMD compared with dry AMD was suggested by previous studies [2,9,40]. As our study focused on NVAMD patients, we were not able to evaluate for differences in the magnitude of the association between the *HTRA1* and *LOC387715/ARMS2* variants and the dry and neovascular forms of AMD.

This study also evaluated if variable phenotype and response to therapy among NVAMD patients may be attributable to the chromosome 10q26 SNPs. The *LOC387715/ARMS2* A69S variant was associated with younger age of examination for AMD, but not with other phenotypic characteristics of NVAMD in a recent study [40]. In accordance with that, a trend toward younger age of onset of NVAMD was associated with both the *HTRA1* and *LOC387715/ARMS2* variants in our study. Another observation suggesting an aggressive phenotype in association with the *LOC387715/ARMS2* variant was reported by Brantley and colleagues [10] who proposed that this variant is associated with larger neovascular lesions. A trend toward an association of both *HTRA1* and *LOC387715/ARMS2* variants with larger neovascular lesions was also observed in this study. While association of the *HTRA1* variant with the classic lesion type was described in a French population [26], such association was not identified in another fair-skinned population which was evaluated [10] or in the Israeli population.

Brantley and coworkers did not find an association between *LOC387715/ARMS2* A69S variant and response to bevacizumab injections in 86 patients. The same group did not find an association between this variant and response to PDT in 69 patients [10,25]. Yet, these authors found an association between response to bevacizumab injections and the CFH Y402H polymorphism. We confirmed this observation with respect to the *LOC387715/ARMS2* variant and expanded it to include the *HTRA1* variant in 143 Israeli NVAMD patients. Both variants were not associated with the visual outcome or with the number of PDT sessions required. Combined, these data do not support the existence of a major pharmacogenetic interactions among chromosome 10q26 SNPs and current therapies for NVAMD.

It is still unclear if the *HTRA1* variant or the *LOC387715/ARMS2* variant has a causative role in the pathogenesis of AMD. While Dewan and Young and their colleagues [7,8] suggested that the *HTRA1* variant is more strongly associated with the disease, Kanda and colleagues [6] suggested that *LOC387715/ARMS2* rather than the *HTRA1* variants explain the association of the chromosome 10q26 region with the disease. Since the variants in these genes are in almost complete linkage disequilibrium in each of the populations reported so far, additional functional studies are required to determine which variant is involved in the pathogenesis of the disease.

In that respect, conflicting evidence has been reported regarding the functional significance of the *HTRA1* promoter variant. Yang and colleagues [7] observed that this variant is associated with increased expression levels of the *HTRA1* gene in 3 AMD patients homozygous to G allele of the *HTRA1* promoter variant compared with 3 control patients homozygous to the A wild-type allele. Dewan and colleagues [8] described increased *HTRA1* expression associated with the promoter variant. These authors speculated that altered *HTRA1* expression levels might be involved in AMD. However, Kanda and colleagues [6] suggested that the same *HTRA1* variant does not affect *HTRA1* expression levels in cell lines or in the retina. We have also failed to identify an association between the *HTRA1* variant and *HTRA1* mRNA levels in WBCs. Yet, this data does not preclude an effect of the same promoter variant on retinal expression levels of *HTRA1* in the context of AMD.

It is still unclear which of the chromosome 10q26 variants is involved in the pathogenesis of AMD. However, our data show that additional genetic and environmental factors which underlie variable phenotype and response to therapy in NVAMD are yet to be identified.
